# Dietary Flavonols Intake and Risk of Esophageal and Gastric Cancer: A Meta-Analysis of Epidemiological Studies

**DOI:** 10.3390/nu8020091

**Published:** 2016-02-16

**Authors:** Yan Xie, Shifeng Huang, Yuxi Su

**Affiliations:** 1 Stem Cell Biology and Therapy Laboratory, Lab medicine of Maternity and Child Care Hospital of Yubei District, Ministry of Education Key Laboratory of Child Development and Disorders, the Children’s Hospital of Chongqing Medical University, Chongqing 400014, China; xieyan1st@gmail.com; 2 Orthopedic Department II, Chongqing Key Laboratory of Pediatrics, Ministry of Education Key Laboratory of Child Development and Disorders, the Children’s Hospital of Chongqing Medical University, Chongqing 400014, China; huangshifeng09@163.com

**Keywords:** flavonols, esophageal and gastric cancer, meta-analysis, epidemiology

## Abstract

*Background:* Esophageal cancer (EC) and gastric cancer (GC) are common cancers and leading causes of cancer deaths worldwide. Many studies have investigated the association between dietary flavonols intake and the risk of EC and GC, but the results are inconsistent. Hence, we conducted a systematic analysis of relevant population-based studies to assess the association and derive a more precise estimation. *Methods*: The Cochrane, PubMed and Embase databases were searched to identify articles published through January 2016 that met the predetermined inclusion criterion. Twelve studies involving 4593 patients and 519,378 controls were included. *Results*: The summary odds ratios (ORs) of EC, GC and the two combined were respectively 0.88 (95% CI: 0.73–1.08), 0.80 (95% CI: 0.70–0.91) and 0.83 (95% CI: 0.74–0.92) for the highest category of dietary flavonols intake compared with the lowest. No significant heterogeneities were observed in these studies. Further analysis showed that the pooled ORs of EC and GC for cohort, population-based case-control and hospital-based case-control studies were 0.90 (95% CI: 0.61–1.34), 0.92 (95% CI: 0.72–1.18), 0.68 (95% CI: 0.38–1.24) and 0.83 (95% CI: 0.65–1.06), 0.84 (95% CI: 0.45–1.59), 0.70 (95% CI: 0.56–0.88). The subgroup analyses revealed a significant association of flavonol intake with a reduced risk of noncardia gastric adenocarcinoma but not gastric cardia adenocarcinoma. Moreover, significant inverse associations of flavonol intake with GC risk were observed in women but not in men, in smokers but not in nonsmokers, in European populations but not in American populations. Similarly, a significant inverse association of flavonols intake with EC risk was also observed in smokers but not in nonsmokers. *Conclusion*: High intake of dietary flavonols is significantly related to a reduced risk of GC, especially in women and smokers.

## 1. Introduction

Esophageal cancer (EC) and gastric cancer (GC) are, respectively, the eighth and the fourth most common cancer and leading causes of cancer deaths worldwide [1,2]. It is worth noting that over the past two decades, esophageal adenocarcinoma (EAC) and gastric cardia adenocarcinoma (GCA) have been among the most rapidly increasing cancer types in the United States and many other Western countries [3,4]. EC and GC, especially EAC and GCA, are often considered to be similar clinical entities because they are both epithelial cancers originating in or near the gastroesophageal junction and have similar five-year survival rates [5]. Previous reports have suggested the intimate association between diet and the risk of cancers. Because the alimentary tract contacts and interacts with dietary components directly, EC and GC, as well as colorectal cancer, may be more closely related to diet. The high consumption of meat, especially red and processed meats, saturated fat, salt and salted food may increase the risk of EC and GC, whereas the consumption of vegetables, fruit and carotene may decrease the risk [6,7,8]. Therefore, the identification of modifiable risk factors, particularly in the diet, for EC and GC is important because it may lead to potential prevention opportunities.

High fruit and vegetable consumptions are associated with beneficial health effects, and these effects have been partly attributed to flavonoids, a group of polyphenolic compounds, which occur ubiquitously in plant foods. Dietary flavonols, mainly including quercetin, myricetin and kaempferol, are a subclass of flavonoids in a daily diet. Dietary flavonols mostly exist in black tea, onions, broccoli, beer, apples, wine and mixed salads in western country diets [9,10]. In recent decades, studies have suggested that dietary flavonols, such as quercetin and myricetin, may be potent anti-carcinogenic substances. Flavonols seem to be effective *in vitro* anti-proliferative and pro-apoptotic agents in a series of tumor cells. Quercetin and myricetin can impact the etiology of certain diseases ranging from oxidative stress and inflammation to carcinogenesis and cancer progression [11,12]. Epidemiological studies have been performed to estimate the association between dietary flavonols and various types of cancer, including EC and GC. However, it is difficult to assess the effects of dietary flavonols on EC and GC. As mentioned above, hot beverages, wine and beer are important sources of dietary flavonols in western country diets, yet hot beverages and alcohol intake are risk factors for EC and GC. Therefore, diet patterns with high intake of flavonols might be associated with high risk of EC and GC. In fact, population-based studies have reported inconsistent findings for the role of dietary flavonols in EC and GC incidence. To derive a more precise estimation of the relationship between dietary flavonols intake and the risk of EC and GC, we performed a meta-analysis to summarize the available evidence from prospective and case-control studies.

## 2. Methods

### 2.1. Search Strategy

A systematic search of the literature published through 2 January 2016, was conducted using the Cochrane, PubMed and Embase databases. The following search terms were used: “flavonoid”, “flavonoids”, “flavonols”, “flavonol”, “quercetin”, “myricetin”, “kaempferol”, “gastric cancer”, “stomach cancer”, “esophagus cancer” and “oesophageal cancer” ((Flavonoid OR flavonoids OR flavonols OR flavonol OR quercetin OR kaempferol OR myricetin) AND (gastric cancer OR stomach cancer OR oesophageal cancer OR esophagus cancer)). We also performed a manual search via reference lists. Only full-length journal articles with a prospective cohort, population-base case-control (PBCC) or hospital-based case-control (HBCC) study design were considered.

### 2.2. Study Selection

Articles were eligible for the present meta-analysis if they conformed to the following criteria: (i) the study design was a population-based study, including a cohort, PBCC or HBCC study; (ii) a relatively complete assessment of dietary flavonols intake was performed; (iii) the association of dietary flavonols intake with the risk of EC or GC was specifically evaluated; and (iv) the relative risk (RR), hazard ratio (HR), or odds ratio (OR) and the corresponding 95% confidence interval (95% CI) values were available. 

### 2.3. Data Extraction

The data from each study and article fulfilling the inclusion criteria were extracted carefully by two independent reviewers. The following information from each article was recorded: (i) the first author’s name and publication year; (ii) the country of origin, the follow-up time used in the study and the study design (prospective cohort study, PBCC study or HBCC study); (iii) the population (numbers of cases and controls); (iv) baseline intake of flavonols (mediam, mg/d); (v) dietary flavonols included in the dietary assessment; (vi) the level of dietary flavonols intake (mg/day); (vii) the RR or OR values from the most fully adjusted model and their 95% CI values; (viii) the listed confounders adjusted for the multivariate analysis. In addition, due to the low incidence of EC and GC, the OR was assumed to be the same as the RR, and the summary results were reported as OR for simplicity [13].

### 2.4. Statistical Analysis

The summary ORs and corresponding 95% CIs of the included studies were used as a measure to assess the association of dietary flavonols intake and the risk of EC and GC. We estimated between-study heterogeneity in each meta-analysis by the Q test and *I*^2^ statistics. Heterogeneity is considered to be significant for *p* < 0.10 or *I*^2^ > 50%. Summary ORs calculations used the Mantel-Haenszel fixed-effects model [14] and the Der Simonian and Laird random-effects model [15,16]. In the absence of between-study heterogeneity, the two methods provide almost identical results. Since fixed-effects models assume that all studies aim at evaluating a common truth and results differ by chance alone. If no obvious heterogeneity existed, the fixed-effects model was selected to pool the data; otherwise, random-effects models are preferable. When statistical heterogeneity was detected, a sensitivity analysis was performed to explore potential sources of heterogeneity, both in the overall pooled estimate and within the subgroups. The potential publication bias was examined by the funnel plot and Egger’s test (*p* < 0.10). All of the analyses were performed using STATA version 11.0 (Stata Corp, College Station, TX, USA). A *p* value < 0.05 was considered to be statistically significant unless otherwise specified. 

## 3. Results

### 3.1. The Characteristics of the Included Studies 

**Figure 1:**
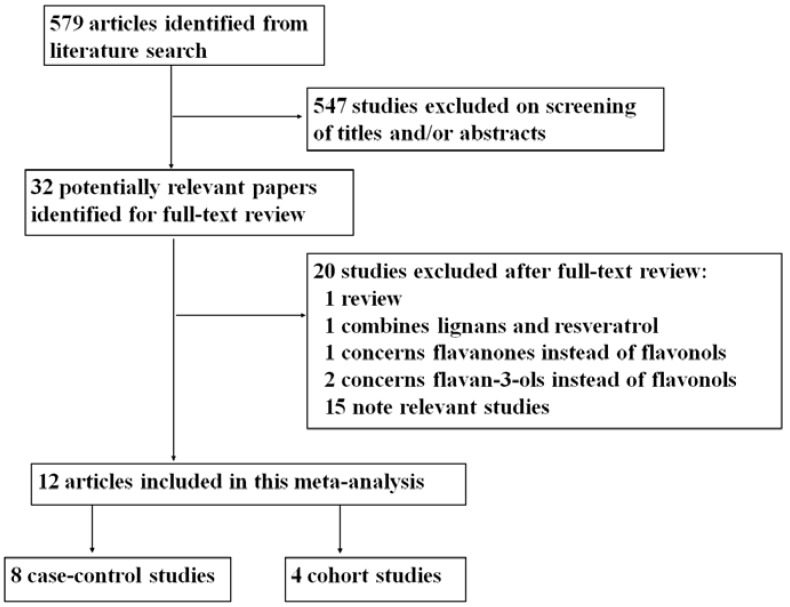
Flow chart showing the study selection procedure.

The present systematic search of the related literatures identified a total of 579 studies. Figure 1 showed the procedure of the study selection. In total, 547 studies were excluded after screening titles or abstracts. The remaining 32 full-text articles were subjected to a more detailed evaluation. Among those 32 studies, 20 were then excluded as irrelevant or because they did not meet the inclusion criteria. Finally, 12 studies relevant to the role of dietary flavonols intake and the risk of esophageal and gastric cancer were included in the present systematic analysis, including four prospective cohort and eight case-control studies [17,18,19,20,21,22,23,24,25,26,27,28]. 

The characteristics of the 12 included studies are shown in Table 1. These selected studies in the final meta-analysis included 4593 cases (1603 EC and 2990 GC) and 519,378 controls. All of the articles were published in English, between 1999 and 2015, a period that spans 16 years.

Among these 12 studies, two were conducted in the USA, one was conducted in Korea, and the other nine were conducted in Europe (one in Sweden, two in Finland, one in Greece, one in Spain, two in Italy and two in 10 European countries). One study only adjusted for age and dietary supplements, and the other 11 studies all adjusted for a wide range of potential confounders of cancer, including age, sex, total energy intake, physical activity, body mass index, smoking status, educational level, alcohol intake, fruit and vegetables consumption, and red and processed meat consumption.

### 3.2. Flavonols Intake and the Risk of Esophageal and Gastric Cancer

**Figure 2:**
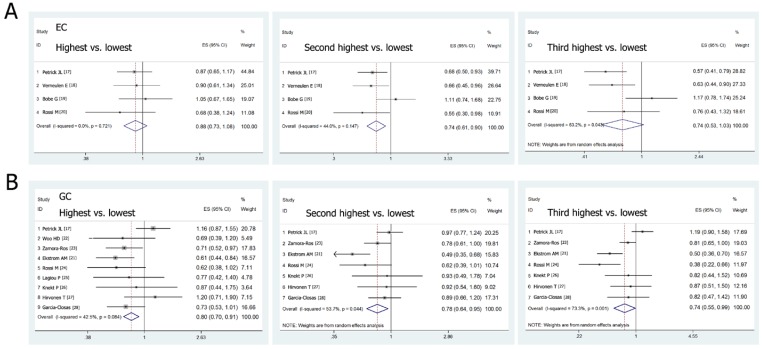
Forest plots investigating the association of dietary flavonol intake and the risk of esophageal cancer (EC) and gastric cancer (GC).

We calculated the summary OR values of EC and GC from the combination of the included studies. Forest plots in Figure 2 showed the summary ORs of EC and GC for the high dietary flavonols intakes versus the low intakes. The meta-analysis data indicated that the pooled ORs of EC, GC and the two combined for the highest category of flavonols intake compared with the lowest category were 0.88 (95% CI: 0.73–1.08), 0.80 (95% CI: 0.70–0.91) and 0.83 (95% CI: 0.74–0.92), respectively. No significant heterogeneities were observed in these studies. The meta-analysis results suggested that the highest intake of dietary flavonols was associated with a reduced, but not statistically, risk of EC, and a significantly inverse association was found between flavonols intake and the risk of GC. Additionally, to assess the effect of the dose of flavonol intake on the association, we selected out the studies in which the levels of dietary flavonol intake were categorized into quartiles. The pooled ORs of EC and GC for the second and the third highest flavonol intake compared with the lowest were 0.74 (95% CI: 0.61–0.90), 0.74 (95% CI: 0.53–1.03) and 0.78 (95% CI: 0.64–0.95), 0.74 (95% CI: 0.55–0.99) respectively. The results suggested that the risk of EC and GC did not decrease with the increasing level of dietary flavonols intake, and no dose-dependent effects were showed. Nevertheless, the high intakes of flavonols were all associated with a reduced risk of EC and GC.

Furthermore, we conducted subgroup analyses by study design, cancer subtype, sex, smoking, population and publication time. As shown in Table 2, the pooled ORs of EC and GC for prospective cohort studies, population-based case-control studies and hospital-based case-control studies were 0.90 (95% CI: 0.61–1.34), 0.92 (95% CI: 0.72–1.18), 0.68 (95% CI: 0.38–1.24) and 0.83 (95% CI: 0.65–1.06), 0.84 (95% CI: 0.45–1.59), 0.70 (95% CI: 0.56–0.88), respectively. No substantial heterogeneities existed across these studies except population-based case-control studies of GC. The subgroup analyses also revealed a significant association for highest dietary flavonol intake with a reduced risk of noncardia gastric adenocarcinoma (NCGA) (OR = 0.73 95% CI: 0.56–0.96), but not gastric cardia adenocarcinoma (GCA) (OR = 1.17 95% CI: 0.82–1.67). Moreover, significant inverse associations between dietary flavonol intake and the risk of GC were observed in women (OR = 0.56, 95% CI: 0.36–0.88) but not in men (OR = 0.96, 95% CI: 0.72–1.27), in smokers (OR = 0.85, 95% CI: 0.73–0.99) but not in nonsmokers (OR = 0.94, 95% CI: 0.76–1.17), in European populations (OR = 0.73, 95% CI: 0.62–0.85) but not in American populations (OR = 1.16, 95% CI: 0.87–1.55), and among the studies published after 2010 (OR = 0.78, 95% CI: 0.67–0.92) but not in those published before 2010 (OR = 0.84, 95% CI: 0.66–1.06). Additionally, a significant inverse association between dietary flavonols intake and the risk of EC was also observed in smokers (OR = 0.73, 95% CI: 0.60–0.90) but not in nonsmokers (OR = 1.25, 95% CI: 0.98–1.58). 

**Table 1:** Characteristics of the included studies.

Author, Year	Region, Period and Design	Cases/Controls	Baseline Intake of Flavonols	Flavonols Included	Flavonols Intake (mg/Day)	RR or OR and 95% CI			Adjustments
Petrick, *et al*. 2015 [17]	USA 1993–1995, PBCC	465/662	Control 14.46 ± 9.41 EAC 14.70 ± 9.68 ESCC 15.74 ± 10.41	Total flavonols	0–8.31 8.32–12.16 12.17–17.81 ≥17.82	EC 1.00 0.57 (0.41–0.79) 0.68 (0.50–0.93) 0.87 (0.65–1.17)	EAC 1.00 0.56 (0.37–0.85) 0.67 (0.45-1.00) 0.80 (0.54–1.18)	ESCC 1.00 0.59 (0.36-0.98) 0.70 (0.43-1.14) 0.97 (0.62-1.53)	Age, sex, race, geographic centre, cigarette smoking, and dietary energy intake.
Verneulen, *et al*. 2013 [18]	10 European countries 1992–2010, Cohort	341 (477312)	Men2 7.1 ± 16.6 Women 27.2 ± 17.6	Total flavonols	Q1 Q2 Q3 Q4	1.00 0.63 (0.44–0.90) 0.66 (0.45–0.96) 0.90 (0.61–1.34)			Age, sex, energy intake, BMI, smoking intensity, educational level, physical activity, alcohol, red and processed meat, fiber, vitamin C and carotenoids.
Bobe, *et al*. 2009 [19]	USA 1986–1989, PBCC	493/1235	W-Con:19.5 W-EAC: 21.0 W-ESCC: 28.6 B-Con: 20.7 B-ESCC: 26.2	Isorhamnetin Kaempferol Myricetin Quercetin	<6.89 6.89–11.0 11.1–15.9 >15.9	1.00 1.17 (0.78–1.74) 1.11 (0.74–1.68) 1.05 (0.67–1.65)	1.00 1.19 (0.66–2.17) 1.26 (0.67–2.38) 0.98 (0.47–2.01)	1.00 1.15 (0.67–1.94) 1.02 (0.59–1.74) 1.10 (0.62–1.97)	Smoking duration and intensity, geographical area, age, BMI, hot tea, hard liquor, beer, red and white wine, caloric intake, education and income.
Rossi, *et al*. 2007 [20]	Italy 1992–1997, HBCC	304/743	22.3	Quercetin Myricetin Kaempferol	≤15.9 16.0–20.4 20.5–25.4 25.5–31.9 >31.9			1.00 1.02 (0.59–1.76) 0.76 (0.43–1.32) 0.55 (0.30–0.98) 0.68 (0.38–1.24)	Age, sex, study centre, education, alcohol consumption, tobacco smoking, BMI and energy intake.
Petrick, *et al*. 2015 [17]	USA 1993–1995, PBCC	589/662	Contro l14.46 ± 9.41 GCA 16.04 ± 10.63 GCGA 14.9 ± 11.36	Total flavonols	0–8.31 8.32–12.16 12.17–17.81 ≥17.82	GC 1.00 1.19 (0.90–1.58) 0.97 (0.77–1.24) 1.16 (0.87–1.55)	GCA 1.00 1.24 (0.81–1.91) 1.01 (0.65–1.57) 1.42 (0.93–2.17)	NCGA 1.00 1.16 (0.80–1.69) 0.96 (0.65–1.14) 0.98 (0.67–1.46)	Age, sex, race, geographic centre, cigarette smoking, and dietary energy intake.
Ekstrom, *et al*. 2011 [21]	Sweden 1989–1995, PBCC	505/1116	NM	Quercetin	0.16–3.88 3.89–6.02 6.03–8.17 8.18–11.9 ≥11.9	1.00 0.68 (0.50–0.93) 0.50 (0.36–0.70) 0.49 (0.35–0.68) 0.61 (0.44–0.84)	1.00 0.39 (0.18–0.83) 0.44 (0.21–0.91) 0.45 (0.22–0.95) 0.76 (0.40–1.44)	1.00 0.76 (0.54–1.06) 0.52 (0.36–0.74) 0.50 (0.34–0.72) 0.57 (0.40–0.83)	Age, gender, socioeconomic status, number of siblings, body mass index, smoking and energy and salt intake.
Woo, *et al*. 2014 [22]	Korea 2011–2014, HBCC	334/334	Control 22.8 ± 19.5 Case 23.3 ± 21.4	Isorhamnetin Kaempferol Myricetin Quercetin	(Median) T1(10.9) T2(14.4) T3(30.8)	GC 1.00 0.86 (0.55–1.36) 0.69 (0.39–1.20)	Men 1.00 0.89 (0.49–1.61) 0.65 (0.32–1.35)	Women 1.00 0.85 (0.37–1.97) 1.22 (0.47–3.16)	Total energy intake, *H. pylori*, age, sex, education, smoking, alcohol, BMI, physical activity, pickled vegetable, red and processed meat, fruits and vegetable.
Zamora–Ros, *et al*. 2012 [23]	10 European countries 1992–2010, Cohort	683 (477312)	Men 26.5 ± 16.4 Women 26.7 ± 17.4	Isorhamnetin Kaempferol Myricetin Quercetin	Q1 Q2 Q3 Q4	GC 1.00 0.81 (0.65–1.00) 0.78 (0.61–1.00) 0.71 (0.52–0.97)	Men 1.00 0.89 (0.66–1.18) 0.81 (0.57–1.13) 0.93 (0.63–1.37)	Women 1.00 0.72 (0.52–0.99) 0.75 (0.51–1.09) 0.45 (0.27–0.75)	Age, educational level, smoking status, physical activity, BMI, alcohol and energy intake, and daily consumption of fruit, vegetables, and red and processed meat.
Rossi, *et al*. 2010 [24]	Italy 1997–2007, HBCC	230/547	22.9 ± 19.1	Total flavonols	≤13.2 13.3–16.4 16.5–20.3 20.3–32.3 >32.3	1.00 0.80 (0.51–1.27) 0.38 (0.22–0.66) 0.62 (0.39–1.01) 0.62 (0.38–1.02)			Sex, age, education, year of interview, BMI, tobacco smoking, and total energy intake.
Lagiou, *et al*. 2004 [25]	Greece 1981–1984, HBCC	110/100	NM	NM	Tper 10 mg	1.00 0.77 (0.42–1.40)			Age, sex, place of birth, BMI, height, years of education, smoking, total energy intake, alcohol, fruits and vegetable.
Knekt, *et al.* 2002 [26]	Finland 1967–1994, Cohort	74(9865)	24.2	Isorhamnetin Kaempferol Myricetin Quercetin	Q1 Q2 Q3 Q4	1.00 0.82 (0.44–1.52) 0.93 (0.49–1.78) 0.87 (0.44–1.75)			Sex, age, geographic area, occupation, smoking, and BMI.
Hirvonen, *et al*. 2001 [27]	Finland 1985–1993, Cohort	111 (27110)	NM	Quercetin Myricetin Kaempferol	(Median) Q1(4.2) Q2(6.7) Q3(9.6) Q4(16.3)	1.00 0.87 (0.51–1.50) 0.92 (0.54–1.60) 1.20 (0.71–1.90)			Age, dietary supplements of α-tocopherol and β-carotene.
Garcia-Closas, *et al*. 1999 [28]	Spain 1987–1989, HBCC	354/354	9.0 ± 7.3	Quercetin Myricetin Kaempferol	Q1 Q2 Q3 Q4	1.00 0.82 (0.47–1.42) 0.89 (0.66–1.20) 0.73 (0.53–1.01)			Total energy intake, intake of nitrites, nitrosamines, vitamin C, total carotenoids and other specific flavonoids.

BMI: body mass index; 95% CI: 95% confidence intervals; EC: esophageal cancer; EAC: esophageal adenocarcinoma; ESCC: esophageal squamous cell carcinoma; GC: gastric cancer; GCA: gastric cardia adenocarcinoma; NCGA: noncardia gastric adenocarcinoma; HBCC: hospital-based case-control; PBCC: population-based case-control; W-Con: White-control; W-EAC: White-EAC; W-ESCC: White-ESCC; B-Con: Black-control; B-ESCC: Black-ESCC; NM: not mentioned.

**Table 2:** Summary estimates of dietary flavonols (highest *vs.* lowest) and esophageal and gastric cancer risk.

Study	EC				GC			
	*n*^a^	RR (95% CI)	Heterogeneity		*n*^a^	RR (95% CI)	Heterogeneity	
			*P*^b^	*I*^2^ (%)			*P*^b^	*I*^2^ (%)
Design								
Cohort	1	0.90 (0.61–1.34)	-	-	3	0.83 (0.65–1.06)	0.208	36.2
Case-control	3	0.88 (0.70–1.10)	0.515	0.0	6	0.79 (0.67–0.92)	0.059	53.0
PBCC	2	0.92 (0.72–1.18)	0.493	0.0	2	0.84 (0.45–1.59)	0.004	88.2
HBCC	1	0.68 (0.38–1.24)	-	-	4	0.70 (0.56–0.88)	0.942	0.0
Cancer type								
EAC	2	0.84 (0.59–1.18)	0.630	0.0				
ESCC	3	0.91 (0.67–1.24)	0.491	0.0				
GCA					2	1.17 (0.82–1.67)	0.111	60.7
NCGA					2	0.73 (0.56–0.96)	0.047	74.7
Gender								
Men	0	-	-	-	3	0.96 (0.72–1.27)	0.379	0.0
Women	0	-	-	-	2	0.56 (0.36–0.88)	0.071	69.4
Smoking								
Smokers	2	0.73 (0.60–0.90)	0.847	0.0	2	0.85 (0.73–0.99)	0.149	52.0
Nonsmokers	2	1.25 (0.98–1.58)	0.843	0.0	1	0.94 (0.76–1.17)	-	-
Population								
American	2	0.92 (0.72–1.18)	0.493	0.0	1	1.16 (0.87–1.55)	-	-
European	2	0.83 (0.60–1.15)	0.439	0.0	7	0.73 (0.62–0.85)	0.442	0.0
Asian	0	-	-	-	1	0.69 (0.39–1.20)	-	-
Publication time								
2010–2015	2	0.88 (0.70–1.11)	0.892	0.0	5	0.78 (0.67–0.92)	0.028	63.1
Before 2010	2	0.90 (0.63–1.28)	0.252	23.8	4	0.84 (0.66–1.06)	0.418	0.0

^a^ No. of selected studies; 95% CI: 95% confidence intervals; EC: esophageal cancer; EAC: esophageal adenocarcinoma; ESCC: esophageal squamous cell carcinoma; GC: gastric cancer; GCA: gastric cardia adenocarcinoma; NCGA: noncardia gastric adenocarcinoma; HBCC: hospital-based case-control; PBCC: population-based case-control.

### 3.3. Sensitivity Analysis

No statistical heterogeneities were found across the studies in the overall pooled estimate. Sensitivity analysis was then performed to evaluate the stability of the results, in which each individual study was sequentially dropped. The analysis was conducted by excluding any single study in turn and pooling the OR of the remaining studies. The summary ORs did not substantially change, with a range from 0.85 (95% CI: 0.68–1.06) to 0.91 (95% CI: 0.74–1.13) for EC and 0.73 (95% CI: 0.63–0.84) to 0.84 (95% CI: 0.73–0.97) for GC, for the highest intake category compared with the lowest. 

### 3.4. Publication Bias

**Figure 3:**
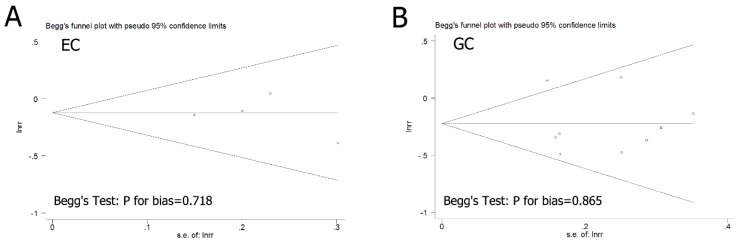
Funnel plot of flavonol intake and the risk of esophageal and gastric cancer.

Begg’s funnel plots and Egger’s tests were performed to assess the potential publication bias in the included studies. As shown in Figure 3, the shape of the funnel plot did not reveal any evidence of apparent asymmetry. Egger’s test, which provides statistical evidence of the funnel plot symmetry, indicated little evidence of publication bias. Therefore, no substantial publication bias was found in these included studies. 

## 4. Discussion

The WHO has indicated that over the next 20 years there will be an approximately 50% increase in new cancer cases attributed to the steadily increasing proportion of elderly people in the world. If the current smoking levels and the adoption of unhealthy lifestyles persist, the increase will be even greater [1,2]. Therefore, the identification of crucial modifiable risk factors in the diet is important for cancer prevention [29,30]. The chemopreventive effects of flavonoids have been extensively investigated in cellular and animal research models; however, the epidemiologic studies assessing the association between flavonoid intake and certain cancer risks have yielded inconsistent results. One potential reason is that different flavonoid subclasses vary in chemical structures and bioactivities. 

The present meta-analysis supports a significant association of dietary flavonols intake with a reduced risk of GC, as well as EC and GC combined, especially in smokers. However, the interpretation of the results should be cautious. The present study has several potential limitations. The main limitation of this meta-analysis is the small number of perspective cohort studies and EC cases included. Especially the number of included studies for subgroup analysis was largely limited, some summary estimates even could not be calculated in several subgroup analyses due to the limited number of studies. So the results, especially subgroup analysis, should be interpreted with caution. More population-based studies are needed to validate the suggestions of the present meta-analysis. Furthermore, another potential confounding factor is the bioavailability of dietary flavonol compounds when estimating the amount of flavonol intake necessary to reduce the risk of EC and GC. In fact, little is known about flavonol absorption in the gastrointestinal tract; the metabolism of flavonols varies by individual, and the degree to which flavonols might have direct effects on epithelial surfaces as they traverse the esophagus and stomach is unclear.

Studies have indicated that dietary flavonol compounds have a variety of bioactivities that may repress carcinogenesis and cancer progression, such as anti-oxidant, anti-inflammation, anti-proliferative and anti-angiogenic activities [31,32,33,34] and may also suppress the effects of cytokines, growth factors, and some crucial enzymes [35,36]. Indeed, quercetin, the main flavonol compound in the daily diet, is able to reduce tumor cell viability, induce apoptosis, and decrease the production of reactive oxygen species via modulation of several key signaling pathways, such as IRE1/JNK, PI3K/Akt, and FOXO3A [37,38]. The chemopreventive effects of flavonols may be exerted by the combination of a series of related bioactivities and would be influenced by many established risk factors for cancer, including smoking status, alcohol consumption, total energy intake, and menopausal status [39,40]. In our study, a significant inverse association of high dietary flavonols intake with a reduced risk of EC and GC was observed in smokers (OR = 0.81, 95% CI: 0.71–0.91) but not in nonsmokers (OR = 1.07, 95% CI: 0.91–1.25). Tobacco smoking can cause oxidative stress, and both oxidative stress and smoking tobacco are related to an increased risk of EC and GC [41,42]. The possible chemopreventive mechanism of dietary flavonols may be attributed to their anti-oxidant properties, not only direct anti-oxidant action, but more importantly the ability to modulate related enzymatic pathways. 

We are unaware of any previous sysmatic analysis of the association for dietary flavonols with risk of EC. The present meta-analysis shows that the pooled ORs of EC for the highest and the second highest flavonols intake compared with the lowest were 0.88 (95% CI: 0.73–1.08) and 0.74 (95% CI: 0.61–0.90), respectively. The results suggested that the higher, but not the highest, flavonols intake may be associated with a significant reduced risk of EC. The phenomenon may be attributed to the reason mentioned before. The diet with highest intake of flavonols tends to have high consumption of hot beverages and alcohol, which are risk factors for EC. Therefore, carefully identifying the sources of dietary flavonols and minimizing the effects of confounding factors are important and essential for evaluating the association between flavonols and cancer risks. Woo and Kim conducted a meta-analysis of GC risk and dietary flavonoids previously; a significant association was found only between flavonols and GC risk based on a limited number of selected studies (OR (95%CI) = 0.68 (0.46–0.99)) [43]. The present study showed similar results. Notably, in our study the pooled ORs of GC for the highest, the second and the third highest flavonols intake compared with the lowest were 0.80 (95% CI: 0.70–0.91), 0.78 (95% CI: 0.64–0.95) and 0.74 (95% CI: 0.55–0.99) respectively. Although all the high intakes of dietary flavonols significantly are associated with reduced risks of GC, the protective effect of dietary flavonols seems decreased with the increasing intake. The phenomenon and the potential reasons might be similar to that in the relation of EC risk and dietary flavonols. 

## 5. Conclusions

In conclusion, the present study indicated a significant association of dietary flavonols intake with a decreased risk of GC, as well as EC and GC combined, especially in smokers. Nevertheless, the results should be interpreted with caution. Residual confounding by alcohol, hot beverages consumption, smoking and total energy intake, etc. is a great concern in studies of EC and GC. Therefore, more carefully designed studies, especially cohort studies, should be conducted to investigate the association of dietary flavonols intake with the risk of EC and GC.
